# Screening for ADHD in Adult Patients With Epilepsy: Prevalence of Symptoms and Challenges to Diagnosis

**DOI:** 10.1177/10870547231197215

**Published:** 2023-09-11

**Authors:** Trung Nguyen, Emily Xiao, Allison Clark, Afroz Shamim, Atul Maheshwari

**Affiliations:** 1 Baylor College of Medicine, Houston, TX, USA

**Keywords:** ASRS, ADHD, seizures, diagnostic barriers

## Abstract

**Objective:**

Given the complex nature of seizure disorders and their treatments, ADHD may be underdiagnosed in this population. We hypothesized that a higher percentage of patients presenting to a seizure clinic would endorse ADHD symptoms compared to rates reported in the general population and that formal screening for ADHD symptoms would identify patients with previously undiagnosed comorbid ADHD.

**Methods:**

In this study, we surveyed 312 adults in a seizure clinic using the Adult ADHD Self-Report Scale (ASRS-v1.1).

**Results:**

We found that 90 patients (28.8%) screened positive with the ASRS-v1.1, but only nine of these patients were able to complete neuropsychological testing,. Out of these patients, only one was diagnosed with possible ADHD.

**Conclusion:**

Through this process, we identified many challenges to making a new ADHD diagnosis in this population, including attention deficits due to other medical or psychiatric diagnoses, a positive urine drug screen, lack of collateral report/information about developmental history, and barriers to neuropsychological evaluation.

## Introduction

ADHD is a neurodevelopmental condition that presents with varying degrees of inattentiveness, hyperactivity, and impulsivity (Magnus et al., 2022). The prevalence of ADHD in the United States is estimated to be 2.1% among young children, 8.9% among school-age children, and 11.9% among adolescents (Danielson et al., 2018). In children, ADHD is associated with significant detriments to mental health and self-esteem (Klassen et a., 2004). The prevalence of ADHD among adults in the US is estimated to be approximately 4.4% (Kessler et al., 2006) and has a strong correlation with depression and anxiety, unemployment, financial stress, and poor well-being (Das et al., 2012).

Epilepsy is a condition typically characterized by recurrent, unprovoked seizures (Fisher et al., 2014). Among children with epilepsy, the prevalence of ADHD is at least 20%, substantially higher than the prevalence in the general pediatric population (Kaufmann et al., 2009). Several mechanisms have been proposed for the association between ADHD and epilepsy in children, including common genetic propensity, shared alterations in neurotransmission, and epileptiform activity directly resulting in lapses in attention (Kaufmann et al., 2009).

In adults with epilepsy, several studies point to a prevalence of up to 20% with comorbid ADHD (Ashjazadeh et al., 2019; Babcock & Ornstein, 2009; Ettinger et al., 2015). The presence of ADHD symptoms in adults with epilepsy is associated with greater psychosocial comorbidities and lower quality of life (Ettinger et al., 2015). Specifically, adults with both epilepsy and ADHD have significantly greater odds of being unable to work due to disability and significantly reduced odds of being employed full time. Furthermore, these patients also experience a ninefold increased incidence of depression and an eightfold increased incidence of anxiety compared to their counterparts without ADHD symptoms (Ettinger et al., 2015). ADHD has a particularly strong association with work-life impairment even after controlling for depression and anxiety symptoms (Das et al., 2012).

Given the higher risk of reduced quality of life, emotional distress, and functional impairments in adults with epilepsy and comorbid ADHD, it is important to efficiently identify and treat these patients appropriately. Effective treatment of ADHD may also improve management of seizure disorders by increasing adherence to medications and/or minimizing precipitating stressors (Brikell et al., 2019). Thus, the objective of this study was to investigate a screening and evaluation procedure for identifying comorbid ADHD in patients who presented to an adult seizure clinic. We hypothesized that a higher percentage of patients presenting to seizure clinic would endorse symptoms suggestive of ADHD compared to rates reported in the general population. We also hypothesized that formal screening for ADHD symptoms in an outpatient seizure clinic followed by neuropsychological evaluation would efficiently identify patients with previously undiagnosed comorbid ADHD.

## Materials and Methods

This was a cohort study of adult patients at a seizure clinic in the Harris Health System in Harris County, Texas. The Harris Health System is the county’s safety net health care entity with a relatively low socioeconomic patient population (da Costa et al., 2022) of 45.9% uninsured, 21.2% on Medicaid/Children’s Health Insurance Program (CHIP) plans, 12% on Medicare and Medicare managed plans, and 19.9% with commercial and other funding. In addition, the racial demographics include a patient population that is 52.9% Hispanic, 24% African American, 14.4% Caucasian, and 8.7% Asian and other, according to publicly available records (Facts and Figures, n.d.). Patients who were seen in this seizure clinic between April 21, 2021 and December 15, 2021 were given the Adult ADHD Self-report Scale-V1.1 (ASRS-v1.1, developed by the World Health Organization) were included in this study (Kessler et al., 2005). Patients who preferred reading in Spanish were given a validated Spanish version of the ASRS-v1.1 (Lozano et al., 2020). The clinic includes a mix of patients with new-onset seizures as well as follow-up for ongoing seizures. The medical record was reviewed for demographic information. A history of ADHD was determined by documentation in the past medical history or being prescribed medication specific for ADHD. The protocol for screening and diagnosing patients with ADHD was reviewed by a licensed neuropsychologist and a board-certified psychiatrist.

**Figure 1.**
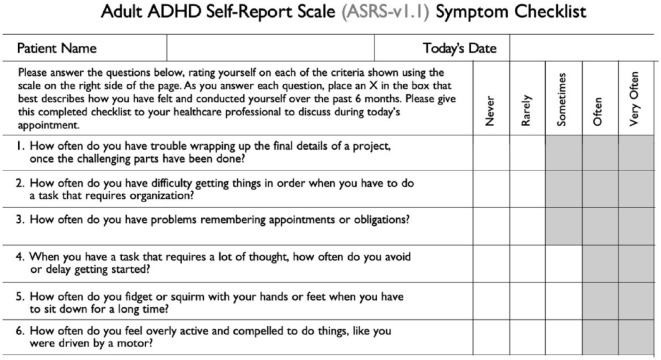
The ADHD Self-Report Scale (ASRS-v1.1) Symptom Checklist (Part A). *Source*. Kessler et al. (2005).

Part A of the ASRS-v1.1 Screener was used to screen adult patients with epilepsy (PWE) for ADHD symptoms (Figure 1). This part of the ASRS-v1.1 consists of six questions, among which the first four questions pertain to inattentive (ADHD-I) symptoms whereas the last two questions pertain to hyperactive (ADHD-H) symptoms. The six-question version of the ASRS-v1.1 is generally preferred over its full version in community surveys (Kessler et al., 2005). Patients who selected the shaded boxes for four or more questions were considered to demonstrate symptoms consistent with ADHD and were interpreted as a positive screening result.

Patients who screened positive for ADHD symptoms were asked to perform a urine drug screen (UDS). Patients with a positive UDS were excluded from the study given the possibility of drug-induced attention deficits. Patients who were English or Spanish speakers and with a negative UDS were subsequently referred for neuropsychological evaluation for ADHD.

## Results

**Figure 2.**
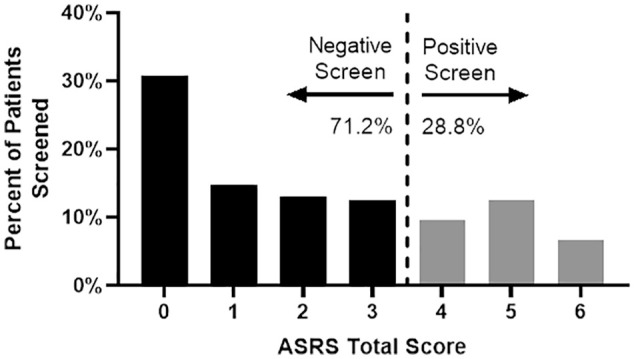
Distribution of total screening scores. Score distribution (0–6) of the ADHD self-reported symptom screen. A score of >4 is a positive screen for ADHD (28.8% of all patients).

We found that 28.8% of patients in our seizure clinic screened positive for ADHD symptoms (90 out of 312 patients; Figure 2). Comparisons between the baseline characteristics of patients who screened positive versus negative for ADHD symptoms revealed no significant differences in age, gender, or number of anti-seizure medications (ASMs). However, patients who identified as White, had an unknown seizure type, or had a history of ADHD were more likely to have a positive screen for ADHD symptoms (Table 1).

**Table 1.** Demographic Characteristics.

	Negative screens (*N* = 222)	Positive screens (*N* = 90)	*p*-Value
Age (mean ± *SD*)	42.6 ± 13.1	45.5 ± 12.3	.072
Gender (*n*, % male)	98, 44.1%	36, 40.0%	.503
Race/ethnicity (*n*, %)			
Hispanic	135, 60.8%	37, 41.1%	.002*
Black	55, 24.8%	30, 33.3%	.124
White	21, 9.5%	19, 21.1%	.005*
Other	11, 4.9%	4, 4.5%	.849
ASRS-v1.1 language (*n*, %)			
English	140, 68.6%	64, 31.4%	.176
Spanish	82, 75.9%	26, 24.1%	
Seizure type (*n*, %)			
Focal	117, 52.7%	35, 38.9%	.001*
Generalized	34, 15.3%	10, 11.1%	.334
Unknown	71, 32.0%	45, 50.0%	.003*
Number of ASMs (mean ± *SD*)	1.6 ± 0.9	1.6 ± 0.9	1.000
History of ADHD (*n*, %)	7, 3.2%	14, 15.6%	<.001*

*Note*. Baseline of PWEs who screened negative and positive respectively for self-reported ADHD. For continuous variables, comparisons made with unpaired *t*-test; for proportions, comparisons made with Chi-square test. *SD* = standard deviation; ASM = anti-seizure medications.

* *p* < .05.

**Figure 3.**
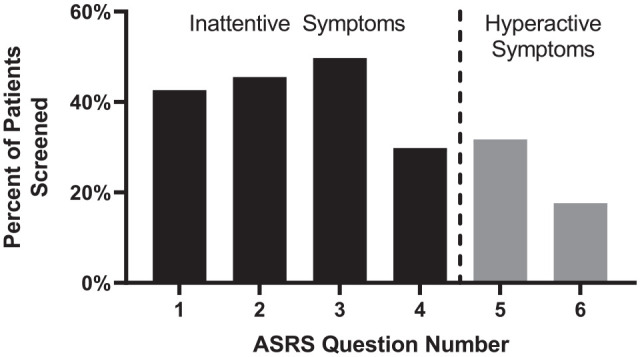
Percentage of patients who met the threshold for screening positive for question items 1 to 6. Significantly greater proportion of patients screened positive for Questions 1 to 4 (inattentive symptoms) than for Questions 5 to 6 (hyperactive symptoms), *p* < .001, Chi-square test.

Additionally, a higher percentage of patients screened positive for inattentive symptoms in question items 1 to 4 (ADHD-I, 41.9% on average) versus hyperactive symptoms in question items 5 to 6 (ADHD-H, 24.7% on average, *p* < .001, Chi-square test, Figure 3).

**Figure 4.**
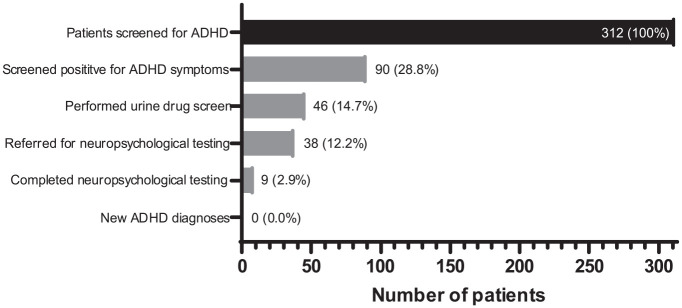
Progression from screening to diagnosis of ADHD. At each step, potential ADHD diagnoses were reduced. After patients screened positive for ADHD symptoms, referral and completion of neuropsychological testing resulted in no new diagnoses of ADHD.

As the 90 patients who screened positive underwent UDS, referral, and neuropsychological testing, the potential for ADHD diagnoses progressively reduced (Figure 4). A total of 46 patients elected to undergo urine drug screening, and 38 of these patients were referred for neuropsychological evaluation. Only nine patients completed the neuropsychological evaluation; none of these patients were formally diagnosed with ADHD, but for one patient, an underlying attentional disorder could not be ruled out. As shown in Table 2, eight of nine patients denied experiencing any attentional symptoms or associated functional impairment since childhood, which is part of the ADHD diagnostic criteria. Five of nine patients had a history of acquired brain injury (ABI), one patient had a possible intellectual disability, and one patient had both an ABI and possible intellectual disability. Of note, 15.6% of patients who screened positive for ADHD had a previous documented diagnosis of ADHD or were previously prescribed ADHD medication, compared to only 3.2% of patients who screened negative for ADHD.

**Table 2.** ADHD Evaluation Results of Nine Patients Who Received Formal Neuropsychological Testing.

Patient	Age (years)	Gender	ADHD history	History of Acquired Brain injury	Intellectual disability	Symptoms or functional problems present prior to age 12 years, by patient or family report	New diagnosis of ADHD per NPT
1	32	F	None	Yes: left frontal AVM	Possible	Denied	No
2	47	M	None	No	Possible	Denied	No
3	60	M	None	Yes: TBI	No	Denied	No
4	53	F	None	Yes: TBI	No	Denied	No
5	61	M	None	Yes: metastases to brain s/p XRT to brain	No	Denied	No
6	37	M	None	Yes: TBI	No	Denied	No
7	60	M	None	Yes: TBI	No	Denied	No
8	62	F	None	No	No	Denied	No
9	45	M	None	No	No	Yes	Possible

*Note*. ASM = anti-seizure medications; F = female; M = male; TBI = traumatic brain injury; XRT = radiation therapy; NPT = neuropsychological testing.

## Discussion

In this study, we found that 28.8% of 312 patients in a seizure clinic screened positive for self-reported ADHD symptoms using the ADHD Self-Report Scale (ASRS-v1.1). This is higher than the 4.4% prevalence reported in the general population, which is consistent with our first hypothesis (Kessler et al., 2006). However, there was significant dropout in the subsequent steps after the screening process and only nine patients underwent neuropsychological evaluation. No new cases of ADHD were diagnosed following neuropsychological evaluation. These findings are limited by the small number of patients who underwent neuropsychological evaluation but do not support the routine screening for ADHD in a seizure clinic unless there are mechanisms in place to facilitate ADHD diagnoses more efficiently.

Of the 312 patients screened, 21 (6.7%) had a documented history of ADHD in their medical record, substantially lower than the 18% prevalence of ADHD in adults with epilepsy that has been previously reported in a study with similar sample size (Ashjazadeh et al., 2019). The ASRS-v1.1 screener was efficient in picking up these patients, since 15.6% of patients who screened positive for ADHD symptoms already had a diagnosis of ADHD, while only 3.2% of patients who screened negative had an ADHD diagnosis (Table 1). The ability to pick up potential patients with ADHD (high sensitivity) at the risk of identifying some patients who do not have ADHD (relatively low specificity) validates the ASRS-v1.1 as an effective screening tool (Hines et al., 2012; Van de Glind et al., 2013). Additionally, patients in our clinic reported more inattentive symptoms (ADHD-I) compared to hyperactive symptoms (ADHD-H), consistent with the higher prevalence of the ADHD-I subtype observed in children with epilepsy (Willcutt, 2012).

The percent of patients that screened positive (28.8%) for ADHD symptoms in our adult seizure clinic is in line with prior studies using the ASRS-v1.1 in adult clinic patients with epilepsy in Iran (35.0%), and adults with self-reported epilepsy who were sent a postal survey in the United States (18.4%) (Ashjazadeh et al., 2019; Ettinger et al., 2015). Some of the variability in positivity rates could be attributable to demographic factors. In our cohort, patients identifying as White had a significantly higher likelihood of having a positive screen, while patients identifying as Hispanic had a significantly lower likelihood of having a positive screen. These findings are somewhat consistent with previous literature showing lower rates of ADHD detection in minority racial/ethnic subgroups, which have been posited to stem from several possible factors, including access to care, insurance status, differences in detection or attribution of symptoms, and cultural influences on the perception of ADHD (Chung et al., 2019).

Patients with focal seizures also were more likely to screen negative for ADHD symptoms (52.7% of all negative screens) while those with an unknown etiology were more likely to screen positive for ADHD symptoms (50.0% of all positive screens). One possibility is that focal seizures are more likely to be acquired later in life secondary to a focal injury, which may preclude the neurodevelopmental profile expected from patients with ADHD. In addition, patients with unknown etiologies due to negative MRI and EEG may be more likely to have psychogenic non-epileptic seizures, which we have previously found to have a higher likelihood of screening positive with the ASRS-v1.1 than patients with epilepsy in an Epilepsy Monitoring Unit (EMU) setting (Dunbar et al., 2021).

Importantly, of the nine patients who screened positive for ADHD symptoms and completed neuropsychological testing, 8 (88.9%) did not meet key diagnostic criteria for ADHD. The primary reason for the low diagnostic yield was the absence of attentional symptoms and functional impairments during childhood. The Diagnostic and Statistical Manual of Mental Disorders, 5th Edition (DSM-V) classifies ADHD as a neurodevelopmental disorder, which must have “several inattentive or hyperactive/impulsive symptoms” present before age 12 and associated functional impairment (American Psychiatric Association, 2013), which was denied by all but one of the patients who underwent a clinical interview as part of their formal neuropsychological evaluation. The reliance on self-report is a relative weakness as patients may have had limited awareness of childhood symptoms. Obtaining collateral report and academic records could also aid in diagnosis, but family/friend reports of patients’ childhood functioning were often limited, and academic records or other supplementary materials were not available for review.

It is also important to note that several participants who underwent neuropsychological evaluation had a history of acquired brain injury and two had a possible intellectual disability. The presence of these comorbid factors may have contributed to the endorsement of attentional symptoms on the ASRS, rather than an underlying attentional disorder. These findings also highlight the non-specificity of attentional symptoms in adults, which further complicates ADHD diagnosis in this population. In future efforts to screen for ADHD in adult populations, the ASRSv1.1 can be coupled with brief follow-up questions regarding the nature of symptoms, symptom onset, and functional impairment to improve the value offered by referral for formal neuropsychological evaluation.

Another important challenge in studying the relationship between ADHD and epilepsy in adults is that epilepsy itself, medications used to treat epilepsy, and comorbidities of epilepsy such as depression or anxiety can present with deficits in attention that do not meet diagnostic criteria for ADHD. Several biological and pharmacological factors are known to cause cognitive impairment in epilepsy, which can present as deficits in learning, memory, and attention (Kaufmann et al., 2009). Experimentally inducing epilepsy with pilocarpine in adult rats has been shown to produce deficits in attention and increases impulsivity on a reaction-time task, which is distinct from the neurodevelopmental condition of adult ADHD (Pineda et al., 2014). In addition, therapy with anti-seizure medications (ASMs) is associated with a higher degree of cognitive impairment, especially with polytherapy and treatment with phenobarbital (Kwan & Brodie, 2001). Finally, epilepsy is associated with many psychiatric (e.g., depression), medical (e.g., migraine), and cognitive (e.g., learning disabilities) comorbidities that may contribute to deficits in attention which can be difficult to differentiate from ADHD (Seidenberg et al., 2009). While none of the patients in our study who received neuropsychological testing were diagnosed with ADHD, many were diagnosed with common epilepsy comorbidities, including mood and neurocognitive disorders, which are important diagnoses to acknowledge and address.

Furthermore, there were many non-medical barriers to diagnosing ADHD in this study, including the completion of a urine drug screen, the patient’s ability to complete neuropsychological testing, scheduling challenges, and out-of-pocket costs. These barriers are especially prominent for the predominantly lower socioeconomic patient population in our county clinic. Moreover, ADHD and its comorbidities are associated with an increased risk of substance abuse in adults. Specifically, the transition from substance use to substance dependence occurs at a faster rate in patients with ADHD than patients without ADHD, likely due to patients with ADHD using illicit substances to self-medicate (Wilens, 2004). Therefore, obtaining a negative UDS as a prerequisite for neuropsychological testing may have deterred some of our patients with ADHD from seeking a formal evaluation. Finally, the availability of neuropsychological testing was limited, and 10.5% of patients referred for testing either missed their appointments or had cancellations. Without surveying the participants, we can only speculate about the potential role that cost or convenience played toward completing a neuropsychological evaluation. Further research should explore these questions further.

With the medical and socio-economic barriers to diagnosis, one option is to integrate psychiatric evaluation into the seizure clinic, which may expedite the diagnosis and treatment of ADHD. As a precedent, one study evaluated the diagnosis and treatment of depression in adult patients in this same seizure clinic. Patients were screened using the Neurological Disorders Depression Inventory for Epilepsy (NDDI-E). For positive screens, neurologists in the clinic conducted a semi-structured psychiatric interview following DSM-IV criteria. If patients received a diagnosis of depression, the same physicians would prescribe treatment accordingly (Friedman et al., 2009). By using this dual approach consisting of a screen for self-reported symptoms followed by a focused structured interview, the study found that 89% of those who screened positive for depression were ultimately diagnosed with Major Depressive Disorder (Friedman et al., 2009). Adopting a similar approach for ADHD may help to mitigate the socioeconomic barriers for diagnosis of ADHD in adults. Timely diagnosis of ADHD is particularly important in this population as previous literature has noted that compared to adults with epilepsy alone, adults with comorbid ADHD have a lower quality of life due to family, social, and work-related dysfunction (Ettinger et al., 2015). Therefore, further research is warranted to identify a systems-based approach to streamline the diagnosis and treatment for patients with seizures and comorbid ADHD.

## Conclusions

In summary, while 28.8% of adults in our seizure clinic screened positive for ADHD symptoms using the ASRS-v1.1 screener, most patients did not complete neuropsychological testing due to a multitude of medical, social, and logistical barriers, and only one out of the nine patients that underwent neuropsychological testing received a possible diagnosis of ADHD. Addressing these challenges may lead to a more efficient diagnosis of ADHD in this high-risk population.
